# From leaf to canopy: Inversion of lettuce pigment distribution using hyperspectral imaging technology combined with deep learning algorithms

**DOI:** 10.1016/j.plaphe.2025.100104

**Published:** 2025-09-12

**Authors:** Yue Zhao, Jiangchuan Fan, Xianju Lu, Ying Zhang, Weiliang Wen, Guanmin Huang, Yinglun Li, Xinyu Guo, Liping Chen

**Affiliations:** a College of Information and Electrical Engineering, China Agricultural University, Beijing, 100083, China; b Beijing Research Center for Information Technology in Agriculture, Beijing Academy of Agriculture and Forestry Sciences, Beijing, 100097, China; c China National Engineering Research Center for Information Technology in Agriculture (NERCITA), Beijing, 100097, China

**Keywords:** Lettuce, Chlorophyll, Carotenoids, Attention mechanism, Quantitative analysis

## Abstract

Plant pigment content is a crucial indicator for assessing photosynthetic efficiency, nutritional status, and physiological health. Its spatial distribution is significantly influenced by variety, location, and environmental factors. However, existing methods for measuring pigment content are often destructive, inefficient, and costly, making them unsuitable for the demands of modern precision agriculture. This study proposes a cross-scale, non-destructive detection method for lettuce pigments by integrating hyperspectral imaging (HSI) technology with deep learning algorithms, addressing the limitations of existing techniques in high-throughput and spatial resolution analysis. In this study, we built a multidimensional dataset based on eight different types of lettuce and developed a deep learning model named LPCNet to predict the contents of chlorophyll *a* (Chl a), chlorophyll *b* (Chl b), carotenoids (Car), and total pigment content (TPC) in lettuce. The LPCNet model integrates convolutional neural networks (CNN), bidirectional long short-term memory networks (BiLSTM), and multi-head self-attention (MHSA) mechanisms, enabling automatic extraction of pigment-related key features and simplifying the complex preprocessing and feature selection procedures required in traditional machine learning. Compared to multivariate analysis methods in machine learning, LPCNet demonstrated superior predictive accuracy, with coefficients of determination ($R_{P}^{2}$) of 0.9449, 0.8613, 0.9121, and 0.8476 for Chl a, Chl b, Car, and TPC, respectively. Additionally, by combining the hyperspectral reflectance of lettuce canopies with the leaf-level inversion model, we visualized the spatial distribution of pigment content on the canopy of lettuce, achieving cross-scale analysis from leaf to canopy. This study provides an innovative approach for the rapid and accurate assessment of lettuce pigment content and offers an effective visualization tool for revealing the physiological processes and growth development of lettuce.

## 1 Introduction

Lettuce is a widely cultivated leafy vegetable globally, known for its short growth cycle, strong adaptability, and high nutritional value, making it popular among consumers [1]. Chlorophyll *a* (Chl a), chlorophyll *b* (Chl b), and carotenoids (Cars), as the primary photosynthetic pigments in lettuce, not only give lettuce its typical green appearance, but also play a crucial role in its growth and nutrient accumulation process [2]. Chl absorbs light energy and converts it into chemical energy, promoting carbohydrate synthesis and enhancing the growth, development, and nutrient reserves of lettuce [3]. Car, as natural antioxidants, protect plant cells from oxidative damage and can be converted into vitamin A in the human body, supporting vision, skin health, and immune function [4]. Therefore, pigment content is not only a common indicator for monitoring the growth and yield of lettuce, but also serves as an important indicator for directly assessing its nutritional value and sensory quality. However, traditional chemical analysis methods, such as solvent extraction and chromatography [5,6], although capable of accurately determining the various pigment contents in lettuce, are typically destructive, complex, and inefficient, making them unsuitable for continuous monitoring or rapid detection in large-scale production. Thus, developing a rapid, non-destructive method to assess pigment content in lettuce is of great significance for guiding agricultural production and meeting consumer demands.

With the development of computer vision and digital imaging technologies, the use of spectral imaging technology combined with machine learning methods has become an important means for non-destructive detection of lettuce. These technologies can quickly and accurately detect various nutrients in lettuce, including glucose, fructose, sucrose, vitamin C [7], and essential mineral elements such as nitrogen (N), phosphorus (P), potassium (K), calcium (Ca), magnesium (Mg), and sulfur (S) [8]. Moreover, they can also detect secondary metabolites in lettuce, such as carotenoids, anthocyanins, flavonoids, and phenolic compounds [9], which are significant for both plant growth and human health. Additionally, researchers have continuously optimized and combined machine learning algorithms to apply hyperspectral imaging (HSI) technology to the evaluation of lettuce phenotypic traits and physiological responses. For example, the combination of wavelet transform (WT), mean centering (MC), and competitive adaptive reweighted sampling (CARS) with partial least squares regression (PLSR) effectively evaluated the fresh weight, dry matter content, and moisture content of lettuce plants [10,11]. First derivative (D1), successive projections algorithm (SPA), random forest (RF), and artificial neural networks (ANN) have also been used to detect the physiological responses of lettuce under water stress, pests and diseases, and pesticide effects [[12], [13], [14]]. However, these methods have limitations in high-throughput processing, requiring extensive combinatorial testing during the modeling process to identify the optimal algorithm combination, and lacking end-to-end automated solutions, which restrict their efficiency and stability in practical applications.

The development of convolutional neural networks (CNNs) has provided new solutions for plant spectral analysis. Compared with traditional machine learning methods, deep learning models do not require the complex feature extraction steps and can automatically learn and extract effective features directly from hyperspectral data, thereby simplifying the data analysis process [3,15]. In non-destructive detection of lettuce, end-to-end methods based on deep learning have shown significant advantages, such as high accuracy, speed, and automation. In recent years, researchers have developed various deep learning models for different quality attributes of lettuce, further verifying their effectiveness in hyperspectral detection. For instance, Yu et al. [15] proposed two end-to-end deep learning models, Deep2D and DeepFC, for predicting soluble solid content (SSC) and pH in lettuce, respectively, both demonstrating high prediction accuracy. Xin et al. [16] introduced a deep learning method based on a combination of stacked autoencoders (10.13039/100009464SAE) and least squares support vector regression (LSSVR) for rapid prediction of cadmium residues in lettuce leaves. Zhou et al. [17] utilized a Monte Carlo-optimized wavelet transform stacked autoencoder (WT-MC-SAE) to detect heavy metal lead in lettuce leaves. Zhou et al. [18] proposed a deep learning method combining wavelet transform (WT) and stacked convolutional autoencoders (SCAE) to detect complex heavy metals in lettuce. Yu et al. [6] developed several deep learning models based on hyperspectral data and time-series phenotypes to predict the quality attributes of lettuce under water stress. Overall, deep learning methods not only greatly improve the efficiency of hyperspectral data analysis, but also improve the accuracy of analysis results, and provide more powerful analytical tools for large-scale agricultural production and intelligent inspection through its end-to-end automation solutions.

However, through literature research, we found that existing studies mainly focus on the scale of lettuce leaves, with relatively less research on the scale of the lettuce canopy [11,13,14]. Moreover, the measurement of Chl content often relies on handheld SPAD instruments [3,19], which have significant errors, limiting the accuracy of the analysis. The determination of Car usually depends on costly chemical methods [4], making related studies rare. Additionally, pigment content varies significantly among different leaves and parts of lettuce. Factors such as the single variety type of lettuce and the small sample size can also reduce the generalization ability of the models [20]. To address these issues, this study collected eight different types of lettuce samples, integrating HSI technology with quantitative analysis methods to develop a high-precision end-to-end deep learning model for investigating spatial distribution differences in pigment content across different lettuce types and plant parts. The main contributions of this study are as follows:

1. An end-to-end deep learning model was proposed for predicting the content of Chl a, Chl b, Car, and TPC in lettuce. Compared to traditional multivariate regression methods, this model features a simpler process flow, higher accuracy, and stronger universality.
2. The ability of the model to capture spectral data features and deal with complex dependencies is enhanced through the multi-module combination strategy. Additionally, by comparing the performance of machine learning's multivariate analysis method and different modules of deep learning, the key factors affecting the model performance are further analyzed.
3. Addressing the issue of significant differences in pigment distribution across different varieties and regions of lettuce, HSI technology combined with deep learning methods is used to construct a prediction model for leaves and invert the pigment content of the entire lettuce canopy, achieving spatial distribution visualization and cross-scale analysis of pigment content.

## 2 Materials and methods

### 2.1 Overview

**Fig. 1:**
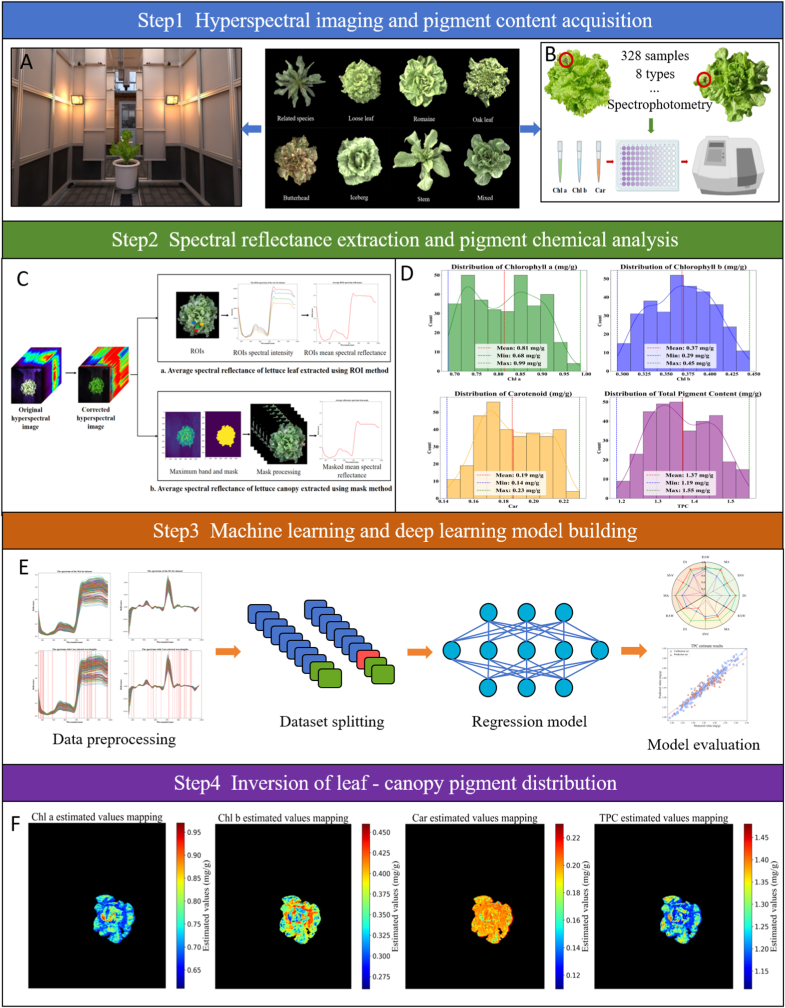
Overall framework of the experiment. (A) Hyperspectral acquisition environment. (B) Measurement of lettuce pigment content. (C) Extraction of spectral reflectance. (D) Statistical analysis of pigment content distribution. (E) Modeling process for pigment inversion. (F) Visualization of pigment distribution. Note: Chl a: Chlorophyll *a*; Chl b: Chlorophyll *b*; Car: Carotenoids; TPC: Total pigment content. Unit: mg/g.

Fig. 1 illustrates the overall process of the experimental design and data analysis used in this study, including lettuce sample types, hyperspectral image acquisition, pigment determination methods, modeling workflow, and visualization results, with the details of the experiments provided in the following subsections.

### 2.2 Plant materials

The experiments in this study were conducted in the experimental fields of the Beijing Academy of Agriculture and Forestry Sciences (39.95°N, 116.29°E). A total of eight different types of lettuce were planted, including romaine, iceberg, butterhead, loose leaf, related species, oak leaf, stem, and mixed [21]. These sample seeds were collected from various countries, covering all the lettuce types. The lettuce seedlings were planted on ridges equipped with ditches and watered and fertilized using an integrated drip irrigation system. Planting began on March 27, 2023, with weekly monitoring of soil moisture content. Irrigation was performed whenever the moisture content dropped below 0.25 ​cm^3^/cm^3^, maintaining it until it rose back to 0.30 ​cm^3^/cm^3^. During the planting period, environmental data, including temperature, humidity, air pressure, and rainfall, were also collected, as detailed in Table S1. The experiments were conducted from May 10 to 14, 2023, with a total of 328 lettuce samples selected, covering all types. The specific sample replicate numbers and source information are provided in Table S2. Subsequently, these samples were transplanted into pots and transported to a nearby laboratory for HSI.

Before conducting HSI, a marker pen was used to mark the largest and most representative leaf of the canopy for each lettuce plant. Then, HSI of the entire lettuce plant was performed to obtain spectral data. After data collection, the marked leaves were clipped, wrapped in aluminum foil, and the lettuce number was recorded. These leaves were then immersed in liquid nitrogen for at least 10 ​min to prevent pigment degradation and subsequently stored in a −80 ​°C freezer for future use. The leaves were taken out when pigment determination was required.

### 2.3 HSI acquisition and correction

Hyperspectral data collection of lettuce was carried out in an imaging chamber (Fig. 1A). The lettuce samples were transported on a conveyor belt to a fixed position beneath the hyperspectral camera, with a distance of 1.2 ​m between the lettuce canopy and the camera. The HSI system used was based on visible to near-infrared (Vis-NIR) technology, operating in the spectral range of 366–976 ​nm. This system included a high-performance push-broom spectrometer (gaiaffield - v10e, dual alix spectral imaging, China), which provided 256 spectral channels with a spectral resolution of 2.8 ​nm. The imaging equipment consists of a charge-coupled device (CCD) camera with a resolution of 775 ​× ​696 pixels, coupled with a 23 ​mm lens, ensuring high image clarity and precise observation of the plants. The system's lighting component comprises four 50-W halogen tungsten lamps, positioned at a 45-degree angle above the lettuce, to ensure uniform illumination over the imaging area. The entire imaging process was controlled by a computer equipped with SpecView software, with an exposure time of 30 ​ms, resulting in HSI of 775 ​× ​696 ​× ​256 dimensions.

In order to eliminate the effects of light intensity and dark current noise on spectral image quality, it is necessary to perform black and white plate correction on the HSI [22]. The light changes in the image are calibrated by scanning a flat polyvinyl chloride (PVC) plate with 99 ​% reflectivity as a white reference. The dark reference is collected by covering the camera lens, removing the dark current generated by the hyperspectral sensor. The reflectance of the HSI was calibrated using the following formula:

(1) $$R=\frac{R_{raw}-R_{d}}{R_{w}-R_{d}}$$

Where $R_{raw}$ represents the original HSI of lettuce, $R_{d}$ and $R_{w}$ represent the HSI of the dark reference and the white reference, respectively, and R represents the corrected HSI of lettuce.

### 2.4 Spectral data extraction

Average spectral reflectance of lettuce leaves and the lettuce canopy were extracted using the region of interest (ROI) method and mask method, respectively, as shown in Fig. 1C. Using ENVI 5.3 software (ITT Visual Information Solutions, Boulder, CO, USA), draw ROIs at the edge and center of the marked lettuce leaf, and calculate the average reflectance of all pixels within the ROI to obtain the average spectral reflectance of the marked lettuce leaf [15]. For the lettuce canopy, a grayscale image (748.76 ​nm) was selected where the reflectance difference between the canopy and the background was maximized to create a mask. A threshold segmentation algorithm was applied to separate the lettuce canopy from the background [8,10], followed by calculating the average reflectance of all pixels within the segmented area to determine the average spectral reflectance of the lettuce canopy.

### 2.5 Pigment content measurement

The contents of Chl a, Chl b, and Car in lettuce leaf samples were determined using spectrophotometry, based on the specific absorption characteristics of pigments at 665 ​nm, 649 ​nm, and 470 ​nm (Fig. 1B). First, 96 ​% ethanol was used as the extraction solvent. A 0.1 ​g sample of labeled leaf tissue, with the midrib removed, was finely chopped and thoroughly rinsed with distilled water. The sample was then mixed with 1 ​mL of extraction buffer and an appropriate amount of stabilizer, followed by thorough grinding under light-protected conditions. The extract was transferred to a 10 ​mL glass test tube, and the grinding mortar was rinsed with additional extraction buffer. All rinse solutions were combined in the test tube and brought to a final volume of 10 ​mL. The tube was then kept in the dark for 3 ​h until the tissue residue was completely decolorized. The supernatant was collected, and absorbance values were measured. Finally, the pigment concentrations were calculated using the following equations.

(2) $$Chla\left(mg/g\right)=13.95\times \Delta A_{665}-6.88\Delta A_{649}\times V/1000\times W$$

(3) $$Chlb\left(mg/g\right)=24.96\times \Delta A_{649}-7.32\Delta A_{665}\times V/1000\times W$$

(4) $$Car\left(mg/g\right)=\left(\frac{1000\times \Delta A_{470}-2.05\times Chla-114.8\times Chlb}{245}\right)\times V/1000\times W$$

(5) TPC(mg/g)=Chla+Chlb+Car

Where V represents the total volume of the extract (mL), and W represents the sample weight (g).

### 2.6 Model methods

Quantitative analysis of pigment content in lettuce using two mainstream methods, machine learning and deep learning, is depicted in the overall modeling process as shown in (Fig. 1E), with detailed parameters of all algorithms available in Table S3. Before machine learning modeling, data preprocessing and feature selection are usually necessary. Preprocessing methods such as Moving Average (MA) can effectively reduce noise in the data, making the spectral curves smoother [11,22]; Standard Normal Variate (SNV) can be used to correct interferences in spectral data, enhancing the reliability of modeling analysis [16,23]; FD1 enhances features in the data, improving the accuracy of the analysis [15]. Furthermore, in terms of feature selection, techniques such as CARS, SPA, Least Angle Regression (LARS), and Uninformative Variable Elimination (UVE) can effectively reduce data dimensions while retaining key feature information [10]. Unlike machine learning methods, deep learning models typically employ an end-to-end training manner, without preprocessing and feature selection steps, directly learning from the data to output the model results.

In terms of dataset partitioning, methods such as Random, Sample set Partitioning based on joint X-Y distances (SPXY), and Kennard-Stone (KS) influence model performance to varying degrees [22]. The Random method partitions the dataset by randomly selecting samples, preventing model overfitting; the SPXY method partitions the data by considering the distances between samples, ensuring that the calibration and prediction sets are representative; the KS method partitions data by maximizing the distances between samples, ensuring consistent distribution between the calibration and prediction sets.

#### 2.6.1 Machine learning model

In the field of multivariate statistical analysis, common machine learning models are mainly divided into multivariate linear regression models and multivariate nonlinear regression models. PLSR is a classic among multivariate linear regression models. It assumes a linear relationship between independent and dependent variables and fits model parameters by minimizing the sum of squared errors [7,9]. However, in many practical situations, the relationships between variables are often nonlinear, making multivariate nonlinear regression models usually more applicable. RF handles complex nonlinear relationships by constructing multiple decision trees and averaging their predictive outcomes [12]. SVR constructs an optimal hyperplane in high-dimensional space and handles nonlinear data using kernel functions to minimize the deviation between actual outputs and predicted values [24]. Furthermore, Extreme Learning Machine (ELM) rapidly learns and processes nonlinear data by randomly generating the weights and biases of neurons in the hidden layer, particularly suitable for scenarios requiring quick responses [25]. These models exhibit significant variations in performance across different datasets and application scenarios, thus selecting the appropriate model for addressing specific issues is crucial.

#### 2.6.2 Deep learning model

**Fig. 2:**
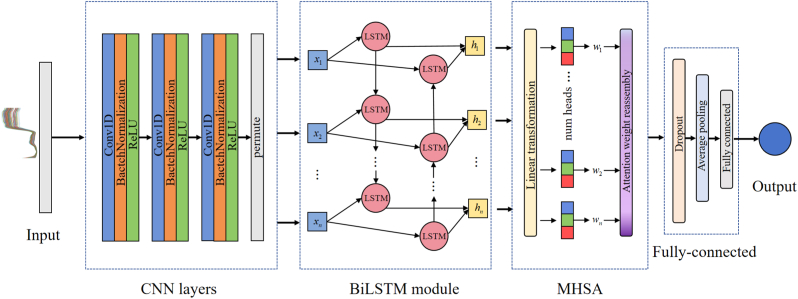
Structure of LPCNet model.

Common deep learning models include CNN, Recurrent Neural Networks (RNN), and their variants [6]. Additionally, numerous studies have demonstrated that attention mechanisms can effectively enhance model performance [26]. In this study, we constructed a deep learning model named LPCNet to perform regression analysis on the pigment content of lettuce. This model integrates CNN, Bidirectional Long Short-Term Memory Networks (BiLSTM) [27], and Multi-Head Self-Attention (MHSA) Mechanisms [28]. The overall structure of the model is shown in Fig. 2, and the structural parameters of the network are presented in Table S4. Firstly, the model uses a three-layer CNN to extract features from the hyperspectral data of lettuce, capturing local dependencies. Subsequently, the BiLSTM layer utilizes its bidirectional structure to capture long-range dependencies in both forward and backward directions within the spectral bands, analyzing the dynamic changes from one wavelength to another. Finally, the MHSA Mechanism is employed to optimize the feature selection process of the model, enhancing performance and generalization capabilities by processing multiple focal points of attention in parallel.

#### 2.6.3 MHSA module

**Fig. 3:**
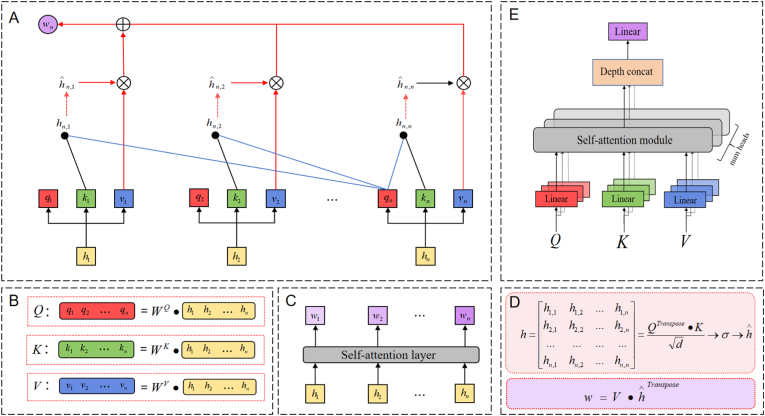
Realization process of the MHSA mechanism. (A) Self-attention mechanism implementation process. (B) Calculation methods for query vectors, key vectors, and value vectors. (C) Weights corresponding to each hidden layer. (D) Total attention weight calculation formula. (E) MHSA mechanism module structure.

Attention mechanisms effectively captures dependencies and key features in data by simulating humans allocate attention when processing information. Self-attention, a critical form of attention mechanism, computes matrices for queries (Q), keys (K), and values (V) of each element in the input sequence and analyzes their interactions to capture dependencies among sequence elements. MHSA, built on the advanced Transformer architecture, enhances the model's data processing capability by handling multiple independent self-attention units in parallel, thus capturing a richer pattern of features [28]. In this study, given the complexity and inter-band correlations of lettuce hyperspectral data, traditional feature extraction methods struggle to accurately capture effective features. To overcome this challenge, we designed an attention module comprising six self-attention units. This module captures complex relationships between bands in lettuce hyperspectral data from multiple perspectives. Specifically, each self-attention unit works independently in different subspaces, learning spectral features related to lettuce pigment content. This approach ensures the comprehensive utilization of multi-dimensional features, thereby enhancing the accuracy of pigment content prediction. By parallel processing multi-dimensional hyperspectral data, model can understand the complex patterns in the data more deeply, achieving higher prediction accuracy and generalization capabilities. The principle of the MHSA module is illustrated in Fig. 3, and the overall embedding process is as follows:

1. For the input hidden layer vector $h_{1},h_{2},\ldots h_{n}$, it undergoes linear transformations through the learned query weight matrix $W^{Q}$, key weight matrix $W^{K}$, and value weight matrix $W^{V}$, resulting in the query vector $q_{n}$, key vector $k_{n}$, and value vector $v_{n}$.
2. For each query vector $q_{n}$, the dot product with all key vectors $k_{1},k_{2,}\ldots k_{n}$ is calculated to obtain the attention scores $h_{n,1},h_{n,2},\ldots h_{n,n}$.
3. The attention scores are normalized using the SoftMax function to obtain the attention weights ${\hat{h}}_{n,1},{\hat{h}}_{n,2},\ldots ,{\hat{h}}_{n,n}$.
4. The weighted sum of the value vectors is performed using the attention weights to obtain the corresponding output vector $w_{n}$ for each query vector.
5. For the weight matrix h of all hidden layers, calculate the total attention weight w.
6. Repeat the above step six times (i.e., six heads), each head with a different linear transformation parameter $W^{Q},W^{K},W^{V}$. Stack the output weights from each head to obtain the output of the MHSA.

(6) $$MultiHead\left(Q,K,V\right)=Concat\left(head_{1},head_{2},\ldots ,head_{n}\right)W^{O}$$

Where $W^{O}$ represents a linear transformation matrix used to convert the output of the MHSA into the output dimensions of the original model.

### 2.7 Evaluation metrics

In order to evaluate the performance of the model, the Root Mean Square Error of Calibration (RMSE_C_), Coefficient of Determination of Calibration ($R_{c}^{2}$), Root Mean Square Error of Prediction (RMSE_P_), Coefficient of Determination of Prediction ($R_{p}^{2}$), and Mean Absolute Error (MAE) are used to assess model performance [16]. The specific calculation formulas are as follows:

(7) $$RMSE=\sqrt{\frac{1}{n}{\sum_{i=1}^{n}\left(y_{i}-\overset{\wedge }{y_{i}}\right)}^{2}}$$

(8) $$R^{2}=1-\frac{\sum_{i=1}^{n}{\left(y_{i}-\overset{\wedge }{y_{i}}\right)}^{2}}{\sum_{i=1}^{n}{\left(y_{i}-\bar{y}\right)}^{2}}$$

(9) $$MAE=\frac{1}{n}\sum_{i=1}^{n}\left|y_{i}-\overset{\wedge }{y_{i}}\right|$$

Where $y_{i}$ is the observed value, ${\hat{y}}_{i}$ is the predicted value, $\bar{y}$ is the mean of the observed values, and n is the number of samples. RMSE reflects the discrepancy between predicted and actual values; a smaller value indicates that the predictions are closer to the actual values, and the prediction accuracy is higher. $R^{2}$ represents the model's ability to explain the variability in the explanatory variables; a value closer to 1 indicates that the model can better explain the variation in the data and has a better fit. MAE is the average absolute difference between predicted and actual values; a value closer to 0 indicates lower prediction error of the model.

### 2.8 Experimental platform

This study uses Windows 10 as the operating platform, equipped with an Intel(R) Core (TM) i7-12700F central processor, and an NVIDIA GeForce RTX 2080Ti to accelerate model training. The system includes 64 ​GB of RAM to ensure smooth data processing. All coding work was completed in a Python 3.8 environment, using libraries including scikit-learn 1.3.0 and PyTorch 1.10.1, among others. Additionally, PyCharm 2019.2.4 (JetBrains s.r.o., Prague, Czech Republic) and Origin 2022 (OriginLab Corporation, Northampton, MA, USA) and RStudio 2023.03.0 (RStudio, PBC, Boston, MA, USA) were used for data analysis and visualization.

## 3 Results and analysis

### 3.1 Quantitative analysis of pigment in lettuce leaves

The quantitative analysis of pigment content in lettuce leaves can effectively characterize their photosynthetic physiological characteristics and varietal differences, providing crucial parameters for quality monitoring. The statistical distribution of pigment content across eight types of lettuce as shown in Fig. 1D, reveal different patterns. Chl a content varied between 0.68–0.99 ​mg/g, exhibiting a significant bimodal distribution pattern. Chl b content displayed a unimodal distribution, primarily concentrated in the range of 0.29–0.45 ​mg/g. Car content followed a right-skewed distribution, with concentrations spanning 0.14–0.23 ​mg/g. TPC approximately adhered to a normal distribution, predominantly aggregating within 1.19–1.55 ​mg/g. These differentiated distribution patterns of pigment content among lettuce types highlight the diversity in the accumulation mechanisms of secondary metabolites across types, laying a robust data foundation for subsequent development of hyperspectral reflectance-based quantitative inversion models for leaf pigment content.

### 3.2 Spectral characteristics analysis of lettuce

**Fig. 4:**
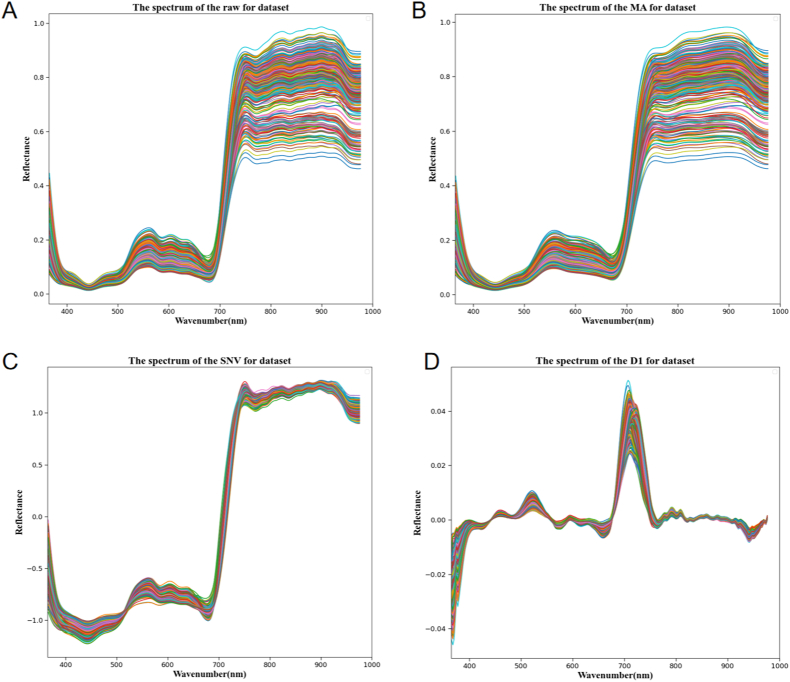
Spectral curves of different types of lettuce and spectral curves after preprocessing. (A) Original spectral reflectance. (B) Spectral reflectance after moving average processing. (C) Spectral reflectance after standard normal variate processing. (D) Spectral reflectance after first derivative processing.

The spectral reflectance characteristics of lettuce reveal its biophysical, physiological, and chemical properties, providing rich information on its growth status [29]. Fig. 4A shows the average spectral reflectance of all types of lettuce across the vis-NIR wavelengths (366–976 ​nm). Within the visible light region, a trough exists between 430 and 470 ​nm, primarily associated with the absorption of lettuce pigments. Chl a (430 ​nm), Chl b (450 ​nm), and Car (470 ​nm) exhibit strong absorption peaks in the blue light region [30], resulting in lower reflectance. Similarly, a significant trough is present at 670–690 ​nm, the absorption peak of Chl a and due to the significant content of Chl a in lettuce, this absorption peak is pronounced [31]. A peak exists near 550 ​nm, representing a typical green light reflectance band where the absorption effect of Chl is minimal. In the near-infrared region, the sharp change in spectral reflectance of lettuce between 690 and 750 ​nm constitutes the “red edge effect” of plants, a result of reduced Chl absorption and cell structure scattering effects [15]. Fig. 4B–D respectively display the average spectral reflectance after preprocessing the lettuce HSI using MA, SNV, and FD1. In the bandwidth regions of 430–470 ​nm, 530–580 ​nm, 670–690 ​nm, and 690–750 ​nm, the positions of peaks and troughs are better highlighted, reflecting the growth status of the plants more effectively. Additionally, the overall trend in spectral reflectance is consistent across the eight types of lettuce. However, the spectral reflectance of different lettuce individuals varies, along with their corresponding pigment content, which indicates that it is feasible to determine the pigment content of lettuce through spectral analysis.

### 3.3 Performance analysis of machine learning models

#### 3.3.1 Performance of different combined models

**Fig. 5:**
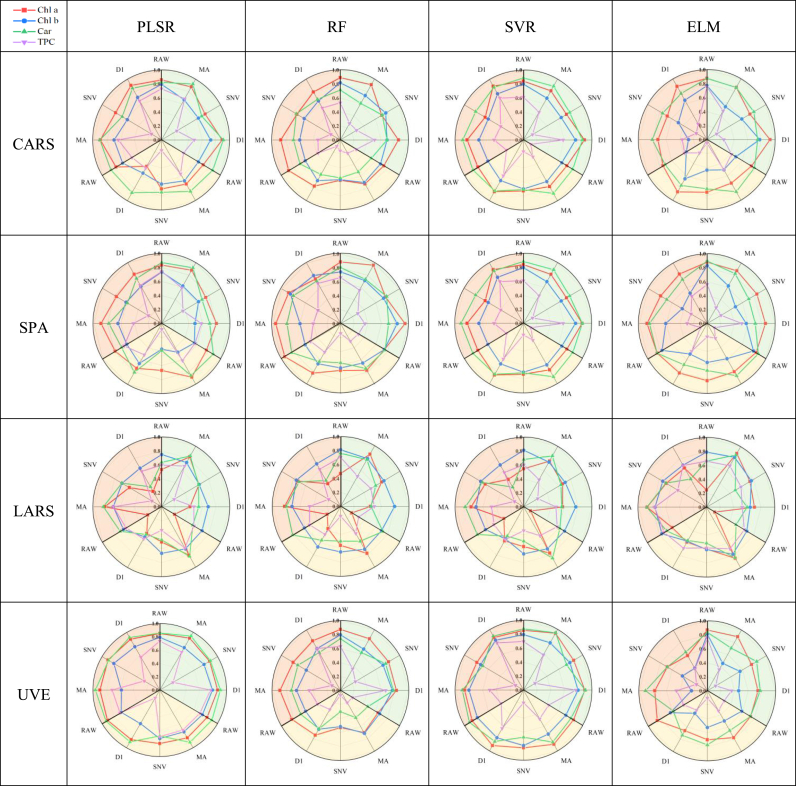
Radar chart of R^2^ performance of machine learning models under different combinations. In the radar chart, each axis represents a different data preprocessing method. A 120-degree sector indicates different dataset partitioning methods. Specifically, the green section represents the SPXY partitioning method, the yellow section represents the Random partitioning method, and the orange section represents the KS partitioning method. Additionally, the lines in the chart represent the performance of different pigment regression models, with the red line indicating Chl a, the blue line indicating Chl b, the green line indicating Car, and the purple line indicating TPC.

Different preprocessing, feature selection, and dataset splitting methods significantly influence the outcomes of machine learning models. To systematically evaluate model performance, we employ radar charts to display the effects of various algorithm combinations. These combinations include no preprocessing and three preprocessing methods, four feature selection algorithms, three dataset splitting methods, and four machine learning regression algorithms. The performance of each combination is assessed based on the R^2^ metric, with experimental results shown in Fig. 5. By analyzing the visualization results of the radar charts, we observed that different preprocessing methods have a significant impact on the performance of machine learning models. Compared to unprocessed data, using MA and D1 preprocessing methods generally enhances model performance. This is because MA and D1 preprocessing effectively reduce noise and baseline drift in spectral data, enhancing the representativeness and stability of the signals [32], thereby improving the predictive accuracy of the model. In terms of feature selection, the CARS method generally shows the better performance. The CARS method utilizes the information of the target variable effectively during the feature selection process, calculating the correlation between spectral features and pigment content in a supervised manner, and selecting the features most related to pigment content, significantly enhancing the model's predictive performance [15]. Additionally, different data set partitioning methods have significant effects on model performance. The results show that the SPXY method outperforms the KS method, and KS method is superior to Random method. This suggests that the SPXY method can maximize the similarity between the calibration and prediction sets while ensuring consistency in data distribution, thus enhancing the model's stability and generalization ability. Different machine learning models perform variably under various processing combinations, and choosing the appropriate model often requires consideration of the specific processing method. Therefore, in conducting machine learning modeling, a comprehensive consideration of preprocessing, feature selection, and dataset partitioning methods should be made to achieve optimal model performance.

#### 3.3.2 Optimal machine learning algorithm combination

**Fig. 6:**
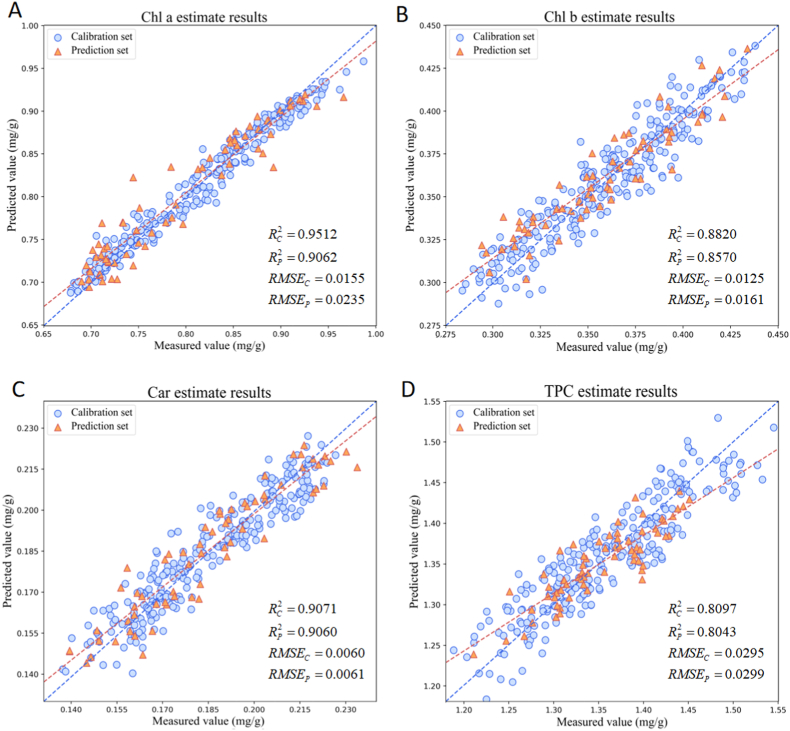
Scatter plots of predictions by optimal machine learning algorithm combinations: (A) Chl a uses the MA-CARS-SPXY-RF algorithm combination. (B) Chl b uses the D1-CARS-SPXY-ELM algorithm combination. (C) Car uses the MA-SPA-KS-SVR algorithm combination. (D) TPC uses the MA-CARS-SPXY-PLSR algorithm combination.

In order to show the prediction performance of the optimal algorithm combination in a more comprehensive way, Table 1 lists the best combination results of each machine learning model in predicting the content of Chl a, Chl b, Car and TPC. Fig. 6 draws the scatter plot of the fitting of the optimal algorithm combination calibration set and prediction set. Through comparison, it was found that different machine learning models showed significant differences in predicting different pigment content. For Chl a, the optimal combination MA-CARS-SPXY-RF achieved a calibration set $R_{C}^{2}$ of 0.9512 and a prediction set $R_{P}^{2}$ of 0.9062, with data points densely distributed near the 45-degree line, indicating a high degree of fit between the predicted and measured values (Fig. 6A). For Chl b, the optimal combination D1-CARS-SPXY-ELM had a calibration set $R_{C}^{2}$ of 0.8820 and a prediction set $R_{P}^{2}$ of 0.8570, with data points similarly closely clustered around the 45-degree line, demonstrating good predictive ability (Fig. 6B). For Car, the optimal combination MA-SPA-KS-SVR resulted in a calibration set $R_{C}^{2}$ of 0.9071 and a prediction set $R_{P}^{2}$ of 0.9060, showing almost consistent performance between the calibration and prediction sets, reflecting high predictive accuracy (Fig. 6C). For TPC, the optimal combination MA-CARS-SPXY-PLSR achieved a calibration set $R_{C}^{2}$ of 0.8097 and a prediction set $R_{P}^{2}$ of 0.8043. Although the predictive accuracy for TPC was slightly lower than for the other prediction models, the data points still aligned well along the fit line, indicating a reasonable level of predictive capability (Fig. 6D). Overall, the prediction results of various pigment contents of lettuce showed high fitting degree and low prediction error, which indicated that the model had good prediction performance and generalization ability.

**Table 1:** Prediction results of pigment content by optimal machine learning algorithm combinations.

Components	Models	$R_{C}^{2}$	RMSE_C_	$R_{P}^{2}$	RMSE_P_	MAE
Chl a	MA-CARS-SPXY-RF	0.9512	0.0155	0.9062	0.0235	0.0176
Chl b	D1-CARS-SPXY-ELM	0.8820	0.0125	0.8570	0.0161	0.0118
Car	MA-SPA-KS-SVR	0.9071	0.0060	0.9060	0.0061	0.0054
TPC	MA-CARS-SPXY-PLSR	0.8097	0.0295	0.8043	0.0299	0.0249

Note: Chl a: Chlorophyll *a*; Chl b: Chlorophyll *b*; Car: Carotenoids; TPC: Total pigment content. Unit: mg/g.

### 3.4 Performance analysis of deep learning model

#### 3.4.1 Performance of the LPCNet model

Deep learning models simplify data preprocessing and feature selection through their end-to-end training and automatic feature extraction capabilities, thereby shortening the data processing workflow [3]. Our developed LPCNet model is capable of learning complex feature representations directly from the raw data, and is used for regression analysis of Chl a, Chl b, Car, and TPC. The loss curve and R^2^ change curve during the training process are shown in (Fig. S1). As can be seen from the figure, the model can reach the convergence state after only a few iterations, and R^2^ is very stable without signs of gradient explosion, which proves the strong fitting ability of LPCNet. The specific experimental results are shown in Table 2. For Chl a, the deep learning model achieved an $R_{C}^{2}$ of 0.9545 on the calibration set; and an $R_{P}^{2}$ of 0.9449 on the prediction set. Compared to the machine learning model, the R^2^ values on the calibration and prediction sets are higher by 0.0033 and 0.0387, respectively, indicating stronger predictive power. Additionally, the deep learning model's MAE is lower, indicating smaller prediction errors. For Chl b, the deep learning model achieves an $R_{C}^{2}$ of 0.9077 on the calibration set, an $R_{P}^{2}$ of 0.8613 on the prediction set, and an MAE of 0.0092. Compared to the machine learning model, the $R_{C}^{2}$ is higher by 0.0257, and the $R_{P}^{2}$ is higher by 0.0043, indicating higher accuracy in fitting training data and stronger model robustness. For Car, the deep learning model achieves an $R_{C}^{2}$ of 0.9161 on the calibration set, an $R_{P}^{2}$ of 0.9121 on the prediction set, and an MAE of 0.0044. Although the R^2^ values of the machine learning model are similar, the lower MAE of the deep learning model indicates smaller prediction errors. For TPC, the deep learning model achieves an $R_{C}^{2}$ of 0.8494 on the calibration set, an $R_{P}^{2}$ of 0.8476 on the prediction set, and an MAE of 0.0190. Compared to the machine learning model, the deep learning model shows the largest increase in R^2^ on both the calibration and prediction sets, higher by 0.0397 and 0.0433, respectively, indicating stronger feature extraction capabilities in capability the relationship between pigment content and spectral reflectance. Overall, the experimental results demonstrate that the LPCNet deep learning model constructed in this study provides higher prediction accuracy and stability in pigment content prediction tasks, reducing prediction errors.

**Table 2:** Prediction results of pigment content by deep learning model.

Components	Models	$R_{C}^{2}$	RMSE_C_	$R_{P}^{2}$	RMSE_P_	MAE
Chl a	LPCNet	0.9545	0.0164	0.9449	0.0180	0.0144
Chl b	LPCNet	0.9077	0.0104	0.8613	0.0111	0.0092
Car	LPCNet	0.9161	0.0056	0.9121	0.0055	0.0044
TPC	LPCNet	0.8494	0.0241	0.8476	0.0237	0.0190

Note: Chl a: Chlorophyll *a*; Chl b: Chlorophyll *b*; Car: Carotenoids; TPC: Total pigment content. Unit: mg/g.

**Fig. 7:**
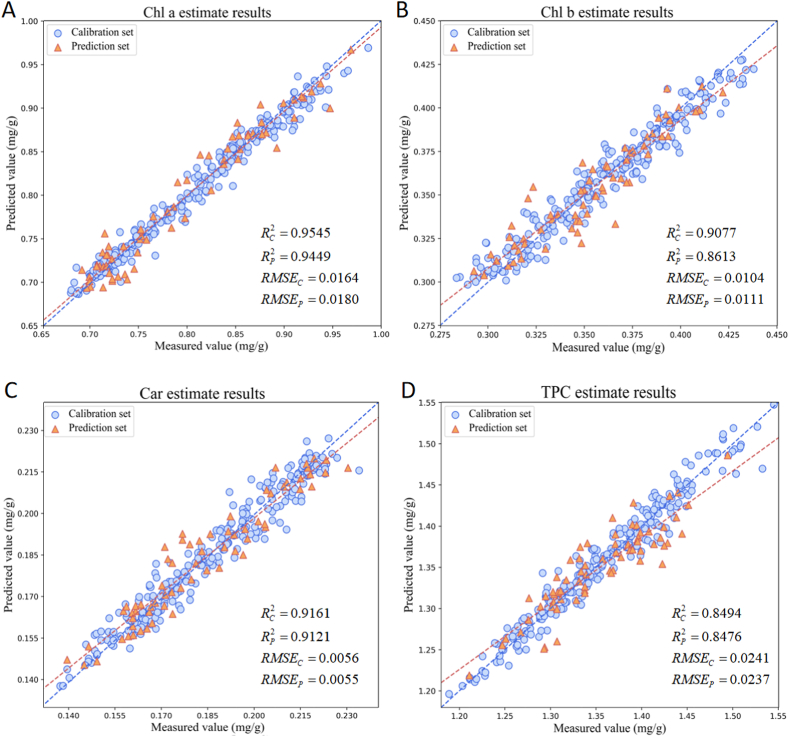
Scatter plots of LPCNet model predictions.

In order to show the prediction performance of the LPCNet model more directly, Fig. 7A–D showed the scatter plots of the prediction results of Chl a, Chl b, Car and TPC, respectively. From the figures, it can be observed that the LPCNet model displays a high degree of fit on both the calibration and prediction sets, with data points tightly distributed and the prediction line close to 45°, indicating high predictive accuracy and consistency. In contrast, although the data points of the previous machine learning model (Fig. 6) are also generally distributed along the 45-degree line, they are overall more dispersed. In particular, the degree of dispersion on the prediction set is greater, indicating that the optimal machine learning combination model is lower than the LPCNet model in terms of prediction accuracy and consistency. Specifically, in the prediction of TPC, the LPCNet model has a tighter scatter distribution and smaller prediction error, which further proves its higher prediction accuracy and robustness when dealing with complex data.

#### 3.4.2 Performance analysis of different modules

To further analyze the performance of the LPCNet model, we conducted ablation experiments on different combinations of three modules: CNN, BiLSTM, and MHSA. To ensure the fairness of the experiments, all models were trained with the same parameters, including convolution kernel size, stride, learning rate, and number of iterations. Table 3 details the performance of CNN, CNN-BiLSTM, CNN-MHSA, and LPCNet on the task of predicting pigment content in lettuce. Using the CNN model as the baseline, the results show that introduction of BiLSTM and MHSA modules can improve model performance to a certain extent. Specifically, the CNN model with the BiLSTM module showed significant improvement in performance on both the calibration set and the prediction set. Particularly in the prediction of Chl b and TPC content, this model even outperformed the LPCNet model on the calibration set, $R_{C}^{2}$ achieving 0.9347 and 0.8569, respectively. However, the BiLSTM model exhibited a large performance difference between the calibration set and the prediction set, with $R_{P}^{2}$ values of 0.8256 and 0.7833, respectively. This phenomenon indicates that while the introduction of BiLSTM improves performance, it also leads to reduced model robustness. On the other hand, the addition of the MHSA module resulted in improved performance in the prediction of Chl a, Chl b, and TPC on both the calibration set and the prediction set, but showed a slight decline in performance for Car content prediction, with $R_{C}^{2}$ and $R_{P}^{2}$ values of 0.8844 and 0.8868, respectively. Notably, the MHSA module showed similar performance on the calibration set and the prediction set for all pigment contents, indicating that the MHSA module can improve the model's performance to some extent while also enhancing its robustness. In summary, the appropriate combination of CNN, BiLSTM, and MHSA modules can improve the model's accuracy for specific tasks while enhancing its robustness. The LPCNet model constructed in this study leverages the feature extraction capabilities of CNN, the forward and backward spectral band feature capture abilities of BiLSTM, and the global information integration capabilities of the MHSA mechanism. This combination achieves more comprehensive, accurate, and robust prediction performance. The LPCNet model demonstrated optimal overall performance in the task of predicting lettuce pigment content, proving the effectiveness of the multi-module combination strategy.

**Table 3:** Performance of different module combinations of the LPCNet model.

Components	Models	$R_{C}^{2}$	RMSE_C_	$R_{P}^{2}$	RMSE_P_	MAE
Chl a	CNN	0.9122	0.0193	0.9078	0.0234	0.0170
	CNN-BiLSTM	0.9506	0.0171	0.9403	0.0188	0.0152
	CNN-MHSA	0.9228	0.0183	0.9258	0.0210	0.0165
	LPCNet	**0.9545**	**0.0164**	**0.9449**	**0.0180**	**0.0144**
Chl b	CNN	0.8356	0.0116	0.8129	0.0131	0.0098
	CNN-BiLSTM	**0.9347**	**0.0088**	0.8256	0.0126	0.0096
	CNN-MHSA	0.8468	0.0114	0.8453	0.0122	0.0095
	LPCNet	0.9077	0.0104	**0.8613**	**0.0111**	**0.0092**
Car	CNN	0.8967	0.0066	0.8915	0.0065	0.0050
	CNN-BiLSTM	0.9022	0.0064	0.8994	0.0065	0.0049
	CNN-MHSA	0.8844	0.0065	0.8868	0.0067	0.0051
	LPCNet	**0.9161**	**0.0056**	**0.9121**	**0.0055**	**0.0044**
TPC	CNN	0.8353	0.0243	0.7393	0.0269	0.0214
	CNN-BiLSTM	**0.8569**	**0.0206**	0.7833	0.0246	0.0198
	CNN-MHSA	0.8425	0.0245	0.8005	0.0241	0.0195
	LPCNet	0.8494	0.0241	**0.8476**	**0.0237**	**0.0190**

Note: Chl a: Chlorophyll *a*; Chl b: Chlorophyll *b*; Car: Carotenoids; TPC: Total pigment content. Unit: mg/g. The results are presented based on the optimal results from 200 iterations.

### 3.5 Visualization of pigment distribution in lettuce canopy

**Fig. 8:**
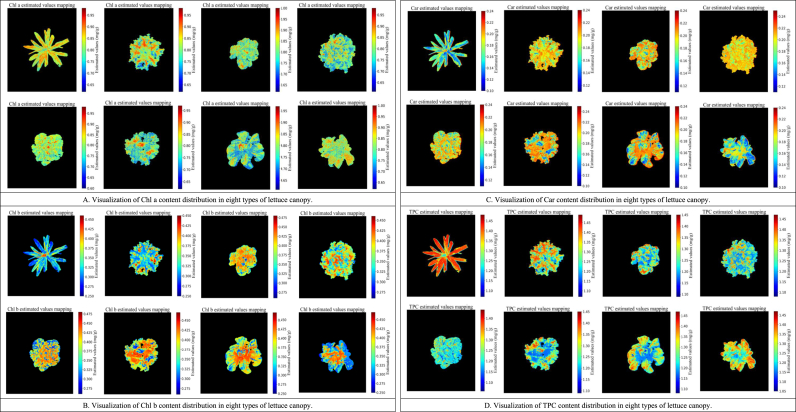
Inversion of pigment content mapping in eight types of lettuce canopy by LPCNet model.

Using the spectral reflectance model of lettuce leaves and combining spectral data of the lettuce canopy, the pigment content of the lettuce canopy can be inferred. Fig. 8 presents the estimated pigment content mapping of each pixel in the lettuce canopy region using the LPCNet model. Different colors and shades represent different pigment contents, with black areas representing the background. As shown in Fig. 8A, the content of Chl a varies significantly in different locations, generally exhibiting an increasing trend from the central area to the periphery. The distribution of Chl a is uneven, with certain specific areas having higher content. This distribution reflects the photosynthetic activity and energy capture capacity of the plant in different parts. The higher content of Chl a in the outer region is closely related to good light conditions, large leaf thickness and high photosynthetic efficiency [7,33]. In Fig. 8B, the distribution of Chl b is opposite to that of Chl a. In areas with high Chl a content, the Chl b content is lower, showing an overall decreasing trend from the outer region to the center. This distribution indicates that Chl b has a more active photoprotective effect outside the canopy [34]. Fig. 8C shows the distribution of Car, which are mostly distributed in the outer parts of the lettuce leaves, showing an increasing trend from the canopy center to the periphery, then decreasing towards the leaf edges. This distribution is related to the role of Car in photoprotection, with the outer regions requiring more Car to withstand stronger light exposure [35]. Fig. 8D displays the distribution of TPC, with higher content at the leaf edges and a general increase from the canopy center to the leaf edges. This reflects the TPC being jointly regulated by various physiological processes, including photosynthesis, photoprotection, and pigment synthesis [36]. Additionally, we found that the distribution of TPC can better depict the texture and venation shape of the lettuce, with areas of different content distributed along the leaf veins, forming distinct texture patterns. Furthermore, we tested multiple quantities of the same type of lettuce. The results indicate that for the same type of lettuce, the pigment distribution is highly consistent (Fig. 1F). However, different types of lettuce exhibit certain variations in pigment distribution, which reflect their distinct environmental adaptation strategies and physiological metabolic characteristics [37]. These false-color images (Fig. 8) visually present the spatial distribution of pigments and their content variations in the canopies of different types of lettuce, which are significant for understanding their photosynthetic characteristics and canopy health status. By analyzing the spatial variations in pigment content among different types of lettuce, we can gain deeper insights into plant physiological processes and the regional differences in photosynthetic activity, providing important references for agricultural production and plant physiology research.

### 3.6 Correlation analysis between lettuce types and pigment distribution

**Fig. 9:**
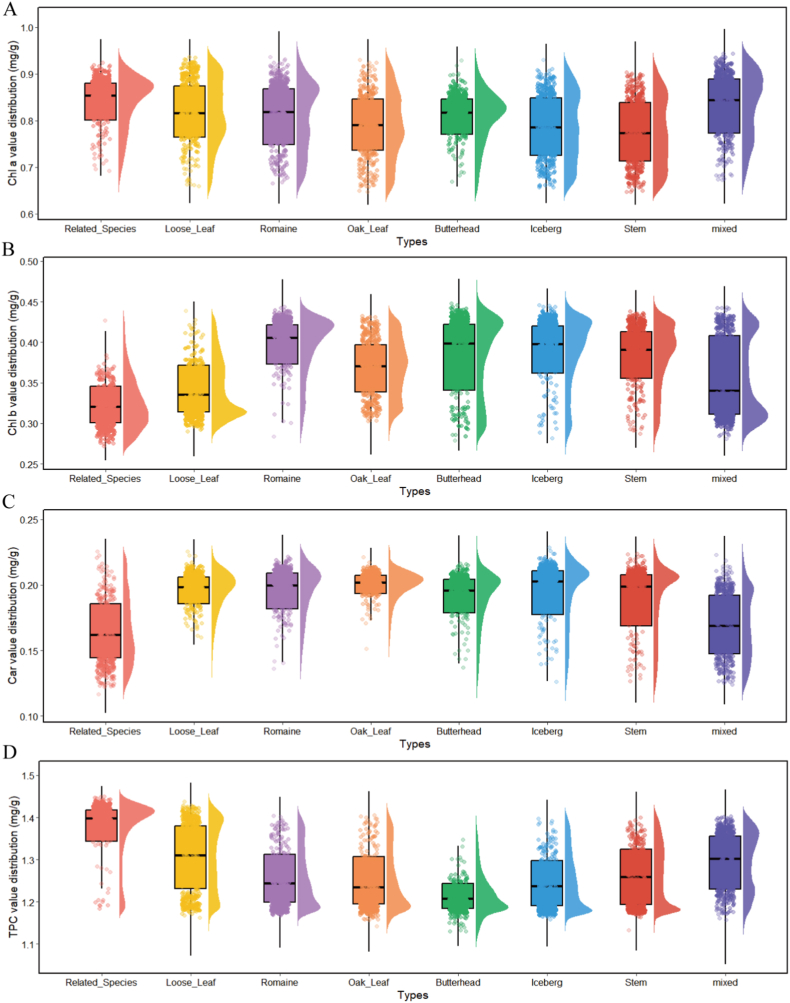
Statistical distribution of lettuce types and canopy pigment content.

Through statistical analysis of canopy pigment contents in eight lettuce types, this study revealed differences in pigment distribution among lettuce types and their underlying regulatory mechanisms (Fig. 9). These variations were closely associated with genetic backgrounds, varietal characteristics, and morphological adaptability. From the distribution characteristics of Chl a (Fig. 9A), it is evident that the Chl a content in Related Species is significantly higher than in other types, such as Oak Leaf, Iceberg, and Stem. This may be attributed to the more active expression of key regulatory genes involved in chlorophyll synthesis in its genotype or to a leaf structure that is more favorable for Chl a accumulation. In contrast, lettuce types such as Oak Leaf, which have relatively lower leaf thickness and canopy light transmittance, may exhibit reconstructed light distribution patterns that impact pigment accumulation efficiency per unit leaf area. The Chl b distribution pattern (Fig. 9B) demonstrated that Romaine lettuce exhibits a typical Chl b enrichment characteristic, while Related Species falls into the lowest-value group. This distribution pattern reflects different light adaptation strategies among lettuce types. Romaine lettuce has thicker leaves, and its chloroplast structure tends to increase the proportion of Chl b to enhance light-harvesting efficiency under low-light conditions. In contrast, lettuce types with lower Chl b content may be more adapted to high-light environments, reducing redundant accumulation of auxiliary pigments to optimize photosynthetic system configuration. Notably, the coefficient of variation for Car among types was significantly lower than that of chlorophylls (Fig. 9C), though Related Species and Mixed still exhibit relatively high distribution dispersion. This phenomenon may relate to genetic heterogeneity in natural hybrids, particularly in cuticle development and photoprotective mechanisms, leading to instability in stress-resistant traits. TPC varied significantly across lettuce types (Fig. 9D), reflecting gene-controlled secondary metabolite synthesis and environmental influences. Overall, the polymorphism of lettuce pigment metabolic network is determined by genetic background, and the differences in genes will directly affect the phenotypic traits of plants and show different environmental adaptability, resulting in different distribution patterns of lettuce pigment. These statistical analysis results provide data support for in-depth understanding of the physiological characteristics of different types of lettuce, and provide theoretical basis for further optimization of lettuce breeding selection and cultivation management.

## 4 Discussion

### 4.1 Comparison of advantages with other pigment detection methods

HSI technology, combined with machine learning and deep learning algorithms, has been widely applied in the assessment of pigment content in lettuce. Compared to existing detection methods, this study demonstrates significant advantages in sample comprehensiveness, pigment determination methods, model accuracy, and pigment distribution visualization. Firstly, this study takes the significant differences in pigment content among different types and parts of lettuce as the starting point. Eight different types of lettuce samples were collected, and the spectral reflectance of leaves and canopies was systematically analyzed. In contrast, other studies often focus on a single cultivar or leaf scale [7,13,19], which limits the sample coverage and may reduce the accuracy and generalizability of the evaluation results. Furthermore, the pigment content of lettuce was determined using spectrophotometric method in this study. This standardized quantitative analysis method is significantly more accurate than the SPAD meter commonly used in other studies [3,19]. Although the SPAD meter is convenient and fast, it can only estimate relative Chl content, introducing considerable errors and making it unsuitable for comprehensive pigment content analysis. Secondly, the LPCNet deep learning model developed in this study automatically extracts features, simplifying the complex preprocessing and feature selection processes required in traditional multivariate analysis methods. The $R_{P}^{2}$ for pigment assessment reaches approximately 0.9. In contrast, previous studies report that Chl content prediction based on CNN and attention mechanisms achieves an $R_{P}^{2}$ of 0.746, while the performance of PLSR and RF models is even lower [3]. Finally, this study achieved the spatial visualization of pigment content within lettuce canopies, enabling a cross-scale analysis from leaf level to canopy level. This is significant for monitoring the pigment distribution of lettuce and assessing its nutritional status, which is an issue not addressed by other studies. In summary, this study demonstrates significant advantages in terms of sample coverage, pigment measurement methods, and model accuracy. Particularly in capturing the diversity of pigment types and spatial distribution, improving the accuracy of deep learning models, and achieving cross-scale visualization from leaf to canopy level, it provides an efficient and innovative solution for non-destructive pigment detection.

### 4.2 Key factors influencing the performance of deep learning models

End-to-end learning is a major advantage of deep learning, as it allows models to directly predict outputs from raw data, reducing potential biases introduced during data preprocessing and feature selection. However, constructing a deep learning model requires the effective combination of different modules [3,6]. Each module serves distinct functions, necessitating researchers to not only select the appropriate base network but also to adjust and optimize the model to suit specific tasks. Although some modules excel in certain functions, their opaque decision-making process remains a challenge. Therefore, understanding and verifying the specific contributions of each module is crucial to ensuring the effectiveness of the model. The experiments in this study demonstrate that the performance of a model can be significantly enhanced by rationally designing and optimizing the combination of deep learning modules. Generally, in experiments where sample sizes are adequate, deep learning modeling is often the better choice. This is because it ensures the model's transferability and generalization capability, and is beneficial for the model's practical application and scalability.

In deep learning applications, the structure, complexity, and parameter settings of the model are crucial factors affecting performance. The design of CNNs, such as the size of the convolutional kernels, stride, and padding, needs to be finely adjusted according to specific tasks to achieve optimal performance [15]. Smaller convolution kernels can extract more detailed features, but they may cause overfitting due to the collinearity of spectral data; larger convolution kernels may overlook important feature information, leading to suboptimal model accuracy. Meanwhile, setting the stride appropriately can capture feature changes more precisely. In building network models, it is common to use multiple convolution kernels of different sizes within the same CNN to capture key features from various perspectives. This approach helps balance the comprehensiveness of feature extraction. Additionally, the setting of model hyperparameters, such as learning rate, number of training epochs, and batch size, requires careful tuning to achieve optimal training results. Overall, a deep understanding and optimization of these key factors affecting model performance can significantly enhance the model's predictive accuracy.

### 4.3 Limitations and future direction

This study developed and validated an LPCNet model for pigment content estimation based on HSI. Through a scientifically rigorous experimental design, it addressed existing issues such as the limited variety of sample types, the lack of consideration for pigment distribution differences, insufficient model accuracy, and the absence of cross-scale analysis and visualization in current research. However, the model still has some limitations when applied to a wide range of practical applications. Because this study was conducted under controlled laboratory conditions, soil moisture and temperature were maintained within ranges optimal for lettuce growth. Under special conditions such as water stress or low-temperature stress, plants' hyperspectral reflectance characteristics exhibit significant changes, posing new challenges for the model's predictive capabilities. Additionally, factors such as plant age, cultivation methods, and environmental conditions (e.g., light intensity, temperature, and humidity) also influence hyperspectral reflectance characteristics, thereby affecting the model's predictive accuracy. These indicate that the model needs to be tested across a broader range of growth stages, under varied cultivation conditions, and in diverse environments to validate its robustness and generalizability.

The core objective of the LPCNet model is to address non-invasive pigment detection challenges. Although this approach is highly suitable for large-scale, high-throughput pigment analyses, it currently does not account for a wider array of agronomic or physiological factors, such as the dynamic response of plant pigments to environmental or management conditions. Integrating these factors into the model requires further data accumulation and interdisciplinary research, combining machine learning and plant physiology to develop a comprehensive model. These limitations highlight the need for further experiments to validate the model's effectiveness under different conditions and to explore its integration with other physiological and agronomic parameters. Addressing these challenges will be a focal point of our future work.

## 5 Conclusion

This study combines HSI technology and deep learning algorithms to develop a deep learning model named LPCNet for non-destructive and high-throughput estimation of pigment content in lettuce. Through experimental analysis of eight different types of lettuce, we successfully achieved cross-scale pigment content inversion and spatial distribution visualization from leaf to canopy. The LPCNet model demonstrated high accuracy in estimating Chl a, Chl b, Car, and TPC with coefficients of determination $R_{P}^{2}$ of 0.9449, 0.8613, 0.9121, and 0.8476, respectively, significantly outperforming traditional multivariate analysis methods. Additionally, by comparing the deep learning multi-module fusion strategy with traditional machine learning multivariate analysis methods, we validated the advantages of deep learning in pigment content estimation, especially in terms of end-to-end operation, automated feature extraction, and high-precision performance. The visualization results revealed the pigment distribution patterns across different types of lettuce, particularly the spatial distribution differences between the leaf and canopy, supporting our hypothesis about the variations in pigment distribution across different types and parts of lettuce. This study provides an effective method for the precise and efficient assessment of lettuce pigment content, which has significant implications for plant health monitoring, agricultural production management, and plant physiological research.

## Authorship contributions

Y.Z.: Conceptualization, Methodology, Visualization, Writing - original draft, Writing - review & editing. J.F.: Methodology, Writing - review & editing. X.L.: Resources, Formal analysis, Data curation. Y.Z.: Investigation, Data curation. W.W.: Conceptualization, Resources. G.H.: Investigation, Resources. Y.L.: Formal analysis, Investigation. X.G.: Funding acquisition, Supervision. L.C.: Funding acquisition, Project administration, Validation.

## Funding

This work was partially supported by the Beijing Rural Revitalization Agricultural Science and Technology Project (NY2401040025), the National Key R&D Program (2022YFD2002300), and the Construction of the Collaborative Innovation Center of 10.13039/501100007934Beijing Academy of Agricultural and Forestry Sciences (KJCX20240406).

## Declaration of competing interest

The authors declare that they have no known competing financial interests or personal relationships that could have appeared to influence the work reported in this paper.

## Data Availability

Further details of the code are available at: https://github.com/zhaoyyy620/spectral_analysis.
