# Supplementary material for: From leaf to canopy: Inversion of lettuce pigment distribution using hyperspectral imaging technology combined with deep learning algorithms
Source: Plant Phenomics. 2025 Sep 12;7(4):100104. doi: 10.1016/j.plaphe.2025.100104 (PMC13109311; doi:10.1016/j.plaphe.2025.100104)
Supplement: Multimedia component 1 [file mmc1.docx]

**From Leaf to Canopy: Inversion of Lettuce Pigment Distribution Using Hyperspectral Imaging Technology Combined with Deep Learning Algorithms**

**Yue Zhao ^1, 2, 3^,** **Jiangchuan Fan ^2, 3^, Xianju Lu** **^2, 3^, Ying Zhang ^2, 3^, Weiliang Wen ^2, 3^,** **Guanmin Huang ^2, 3^, Yinglun Li ^2, 3*^, Xinyu Guo ^2, 3*^, Liping Chen ^1, 2, 3*^**

^1^ College of Information and Electrical Engineering, China Agricultural University, Beijing 100083, China.

^2^ Beijing Research Center for Information Technology in Agriculture, Beijing Academy of Agriculture and Forestry Sciences, Beijing 100097, China.

^3^ China National Engineering Research Center for Information Technology in Agriculture (NERCITA), Beijing 100097, China.

**First Author：**

Yue Zhao:(e-mail: zhaoy@cau.edu.cn)

***Corresponding author:**

Yinglun Li (e-mail: liyingl@nercita.org.cn)

Xinyu Guo (e-mail: [guoxy@nercita.org.cn](mailto:guoxy@nercita.org.cn))

Liping Chen (e-mail: chenlp@nercita.org.cn)

| 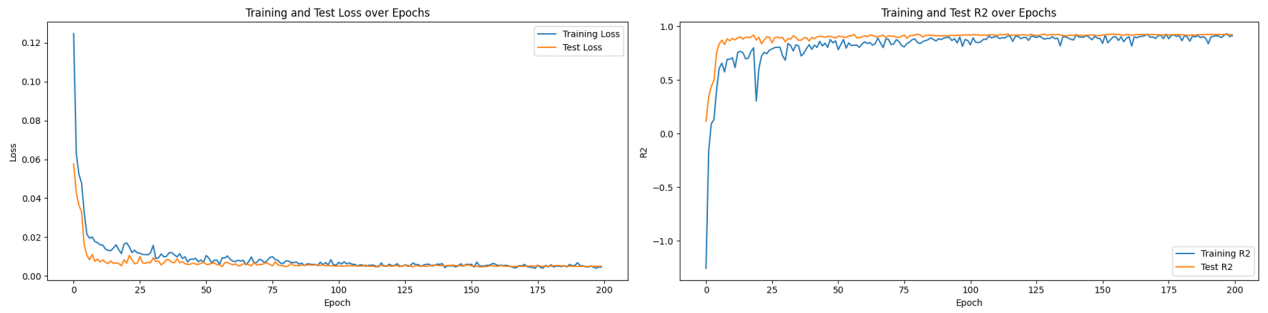 |
| --- |
| 1. Loss curve and R^2^ curve for Chl a |
| 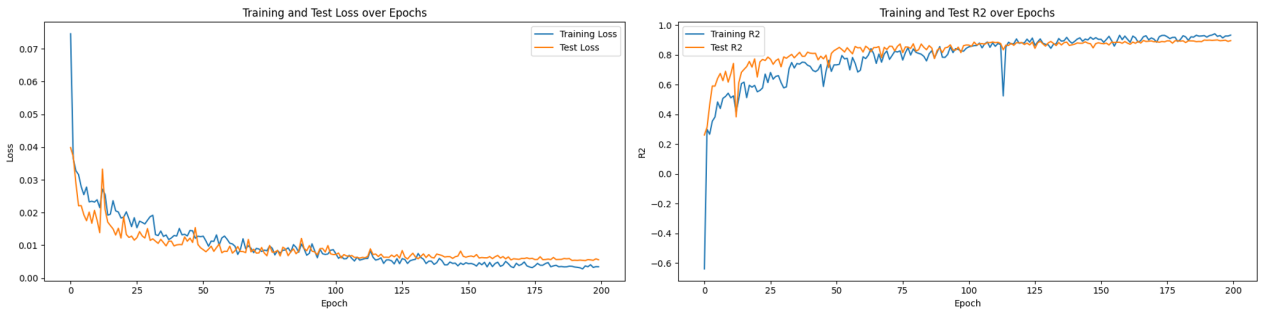 |
| 1. Loss curve and R^2^ curve for Chl b |
| 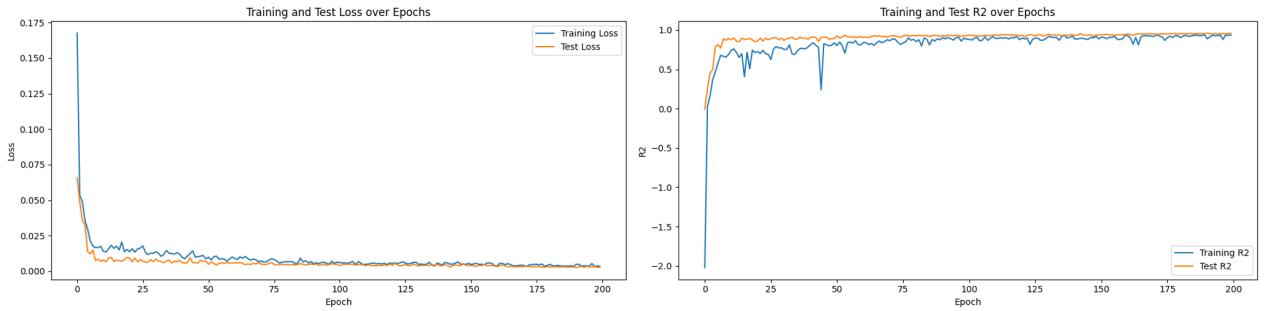 |
| 1. Loss curve and R^2^ curve for Car |
| 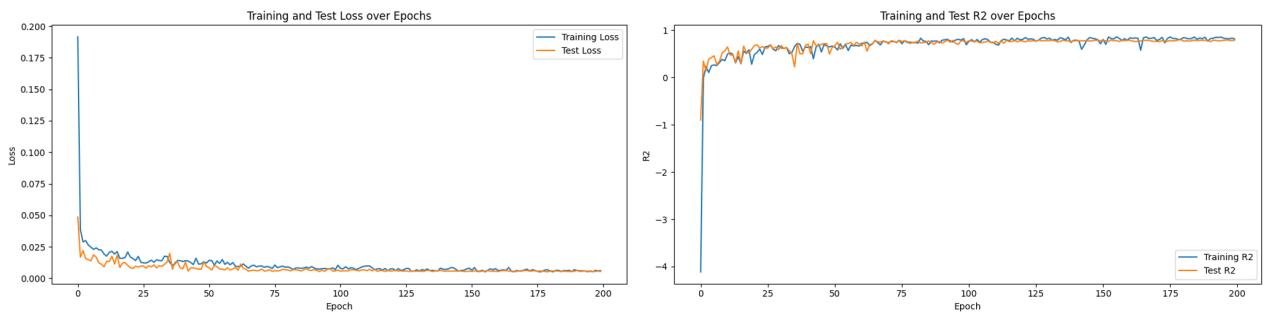 |
| 1. Loss curve and R^2^ curve for TPC |

Fig. S1. Loss curve and R2 change curve of the LPCNet model.

Table S1 Environmental information during lettuce cultivation

| Time | PAR  (µmol/m²s) | | | Relative Humidity (%) | | | Temp  (°C) | | | Pressure  (kPa) | | | Precip  (mm) | | |
| --- | --- | --- | --- | --- | --- | --- | --- | --- | --- | --- | --- | --- | --- | --- | --- |
|  | Min | Mean | Max | Min | Mean | Max | Min | Mean | Max | Min | Mean | Max | Min | Mean | Max |
| D5 | 0 | 260 | 1118 | 0.23 | 0.45 | 0.72 | 8.4 | 18.12 | 25.8 | 100.6 | 101.0 | 101.59 | 0 | 0.00015 | 0.2 |
| D10 | 0 | 148 | 1380 | 0.27 | 0.54 | 0.83 | 7.9 | 13.83 | 18.8 | 100.7 | 101.2 | 101.6 | 0 | 0.00763 | 0.2 |
| D15 | 0 | 328 | 1409 | 0.11 | 0.31 | 0.74 | 4.2 | 15.31 | 26.2 | 99.1 | 100.8 | 101.9 | 0 | 0 | 0 |
| D20 | 0 | 290 | 1391 | 0.12 | 0.33 | 0.73 | 5.0 | 16.72 | 23.9 | 99.7 | 100.5 | 101.2 | 0 | 0 | 0 |
| D25 | 0 | 277 | 1417 | 0.17 | 0.44 | 0.76 | 8.1 | 17.51 | 27.6 | 99.5 | 100.7 | 102.3 | 0 | 0.00014 | 0.2 |
| D30 | 0 | 429 | 1424 | 0.19 | 0.43 | 0.81 | 7.3 | 15.16 | 20.1 | 100.4 | 101.4 | 102.5 | 0 | 0 | 0 |
| D35 | 0 | 431 | 1552 | 0.18 | 0.46 | 0.82 | 10.9 | 19.21 | 26.5 | 100.0 | 100.6 | 100.9 | 0 | 0.00811 | 0.8 |
| D40 | 0 | 248 | 1364 | 0.22 | 0.57 | 0.85 | 14.1 | 20.34 | 28.4 | 100.1 | 101.2 | 101.3 | 0 | 0.00086 | 0.2 |
| D45 | 0 | 392 | 1506 | 0.16 | 0.39 | 0.69 | 12.7 | 21.70 | 29.5 | 100.7 | 101.3 | 101.6 | 0 | 0 | 0 |
| D50 | 0 | 343 | 1534 | 0.26 | 0.58 | 0.83 | 11.7 | 23.92 | 31.2 | 98.9 | 100.1 | 100.8 | 0 | 0.00097 | 0.2 |

Note：PAR (µmol/m²s): Measured by QSO-S PAR sensor; Relative Humidity (%): Measured by VP-4 sensor; Temperature ($^{\circ}$C): Measured by VP-4 sensor; Pressure (kPa): Measured by VP-4 sensor; Precipitation (mm): Measured by ECRN-100 sensor. Each value represents the minimum, mean, and maximum measurements for every 5-day period during the cultivation phase.

Table S2 Types of lettuce and source information

| Types | Countries | Collection time | Numbers |
| --- | --- | --- | --- |
| Romaine | Greece | 1983 | 68 |
| Iceberg | United States | 1994 | 62 |
| Butterhead | Netherlands | 1969 | 44 |
| Loose leaf | France | 1961 | 36 |
| Related species | Turkey | 1983 | 22 |
| Oak leaf | United States | 1989 | 28 |
| Stem | China | 1993 | 24 |
| Mixed | Germany | 1986 | 44 |

Table S3 Detailed parameters of the modeling algorithm

| Types | Methods | Setting parameters |
| --- | --- | --- |
| Spectrum  preprocessing | MA | Window Size = 11 |
|  | SNV | None |
|  | D1 | None |
| Wavenumber select | CARS | Iterations=50, components=20, cross-validation=10 |
|  | SPA | Minimum variables=1, Maximum variables=None, Autoscaling=1 |
|  | LARS | components=30 |
|  | UVE | components=1, repetitions=500,  test_size=0.2 |
| Dataset splitting | Random | test_size=0.2 |
|  | SPXY | test_size=0.2 |
|  | KS | test_size=0.2 |
| Regression model | PLSR | n_components=8 |
|  | RF | n_estimators=600 |
|  | SVR | C=1000, gamma='scale', kernel='rbf' |
|  | ELM | add_neurons(50, 'sigm') |
|  | LPCNet | lr = 0.001, batch_size = 16,  optimizer= 'adam'，epoch=200 |

Table S4 Configuration of LPCNet

| Layer Type | Configuration |
| --- | --- |
| Convolution Layer 1 | 10 @ 1 x 25 (stride=3, padding=0) |
| BatchNorm Layer 1 | BatchNorm1d(10) |
| ReLU Layer 1 | ReLU() |
| Convolution Layer 2 | 15 @ 10 x 15 (stride=3, padding=0) |
| BatchNorm Layer 2 | BatchNorm1d(15) |
| ReLU Layer 2 | ReLU() |
| Convolution Layer 3 | 18 @ 15 x 10 (stride=3, padding=0) |
| BatchNorm Layer 3 | BatchNorm1d(18) |
| ReLU Layer 3 | ReLU() |
| BiLSTM Layer | Input Size: 18 |
|  | Hidden Size: 18 |
|  | Num Layers: 1 |
|  | Bidirectional: True |
| Multi-Head Attention Module | Hidden Size: 36 (2 * 18) |
|  | Num Heads: 6 |
| Dropout Layer | Dropout (0.5) |
| Fully Connected Layer | Input Size: 36 |
|  | Output Size: 1 |
